# Supplementary material for: Barriers and facilitators for the implementation of Antimicrobial Stewardship Programs in Dar es Salaam Regional Referral Hospitals (RRHs)
Source: PLOS Glob Public Health. 2026 Mar 23;6(3):e0006123. doi: 10.1371/journal.pgph.0006123 (PMC13008068; doi:10.1371/journal.pgph.0006123)
Supplement: S1 Text — (PDF) [file pgph.0006123.s001.pdf]

KEY INFORMANT INTERVIEW GUIDE

INTRODUCTION

I would like to take this opportunity to thank you for taking time to participate in this interview. You should be assured that your response will be confidential and that it will only be used for the purpose of this study. Please feel free to answer all questions with utmost openness, and any time you wish to stop the interview, I will be obliged to do as you wish without causing you any consequences.

PARTICIPANT DEMOGRAPHICS

Name of interviewer (Initials)……………………………

Date of Interview………………………..

Name of Hospital……………………….

Participant Number (TEM 01, MWAN 01 or AMA 01)………………………………

Participant's cadre……………………………

Age……………………

Gender………………………..

Highest level of Education…………………..

How long have you worked here?...

INTERVIEW QUESTIONS

1. What do you know about antibiotic stewardship program?

Probe:

· Meaning of antibiotic stewardship

· Responsible persons for antibiotics stewardship

· Activities performed under the umbrella of antibiotics stewardship

· How have you been involved in the implementation of antibiotic stewardship activities

2. Does your facility receive any budgeted financial support for Antimicrobial stewardship programs?

Probe:

Salary, training, resources, mentorship, supervision and Information Technology

3. What is your overall perception or experience of the antibiotic stewardship? Probe: How does antibiotic stewardship impact the quality of delivery of antibiotics? Have you noticed changes in the delivery of antibiotics? How have they changed?

4. Does your facility produce antibiotic susceptibility reports (either monthly or yearly)

   Probe:

Who produces the reports? How is the report used and how is it perceived by medical personnel?

5. Does your facility have facility specific treatment guidelines based on the national guideline to assist with antibiotic selection for common clinical conditions?
6. Does your facility have antibiotics that require pre-authorization before prescribing? Who does the pre-authorization?
7. Does your facility conduct antibiotic reviews and give feedback to prescribers?
8. What do you think facilitates the implementation of ASP at your facility?
9. Are there any challenges that are encountered while implementing ASP at your facility?

Thank you for your participation in this interview. Before we close this interview, do you have any additional information you would like to share with regards to antimicrobial stewardship programs? Or if you have any recommendations on how these programs can be fully utilized in the country?
