# Supplementary material for: Barriers and facilitators for the implementation of Antimicrobial Stewardship Programs in Dar es Salaam Regional Referral Hospitals (RRHs)
Source: PLOS Glob Public Health. 2026 Mar 23;6(3):e0006123. doi: 10.1371/journal.pgph.0006123 (PMC13008068; doi:10.1371/journal.pgph.0006123)
Supplement: S1 Data — (ZIP) [file pgph.0006123.s002.zip › Transcript_2_compressed.pdf]

RESPONDENT (RT)

INTERVIEWER (IR)

IR: As I introduced myself earlier, my name is Berthania Magesa, a master's student at Muhimbili University, pursuing a master's degree in Monitoring and Evaluation in Health. I am currently conducting research to assess the effectiveness of Antimicrobial Stewardship (AMS) teams in reducing the burden of antimicrobial resistance in Dar es Salaam RRHs. I understand that our referral hospitals in Dar es Salaam have been capacitated to establish these AMS programs, and XXX, as one of the hospitals, benefits from these programs.

IR: So, I would like to start with the first question to understand which language you prefer to use.

RT: Swahili.

IR: You are allowed to mix if you wish... Alright, I would like to know your understanding about these Antimicrobial Stewardship programs and when they started at XXX?

RT: These programs at XXX have two aspects. There is a national indirect lab part where they bring QA [quality assurance] to look at resistance. But for our facility, we started following up about two years ago, and it has been effective since last year after we went for training and started the follow-up. Before that, about two or three years ago, I cannot say exactly how many years, but around two or three.

IR: And how are you involved in these programs, in other words, what role do you play in the AMS team?

RT: My role here at XXX, as I told you... first, I am the chairperson of AMS. Before being the chairperson, because I am on this side of the laboratory and I am the main producer of microbiology data, we were doing Antibiogram and also surface toucher to observe the resistance trend. So, to participate, I do it in two ways: as a chairperson, and at the same time, I am in the microbiology department.

IR: Does the institution receive any support in terms of training or allowances for those in the AMS team?

RT: Mhh, no allowances for the AMS team, but we received training and have been trained.

IR: And is there perhaps surveillance supervision?

RT: Surveillance, yes, from the ministry.

IR: How often does it happen? Quarterly?

RT: Let me say quarterly, I don't have the exact... it's quarterly.

IR: And what is your overall view regarding these programs since they started here?

RT: The AMS ones?

IR: Yes.

RT: Ahaaa, my view is still with the doctors because they are the main prescribers of drugs. Sometimes you know when people want to change, they take time. You can provide culture results but the patient has already received drugs.

IR: Okay, has it helped to improve the quality of prescriptions or has it helped to reduce the burden of Antimicrobial Resistance since they started, roughly?

RT: It has increased the burden.

IR: Increased the burden? Even though the programs exist, they are not implemented as required?

RT: Although for our laboratory side, we have a program we want to advance. We even want to invite some people to come for microbiology-related training. The department enabled us to do microbiology sensitization so that they are aware of AMS and such things.

IR: Do you provide reports on antibiotic resistance Antibiogram data?

RT: Now, Antibiogram, I told you we started about three years ago. In the period we were doing it, it wasn't up to standard; we were doing it quarterly, although the number of isolates didn't meet the standards. But we got training, we became more advanced and aware, and we are supposed to prepare Antibiogram. So currently, we are in the phase of collecting those isolates, which we started in November last year, and we are still using traditional methods and global reporting system.

IR: Is this a WHO system?

RT: It's the WHO microbiology system itself that includes patients and those isolates, patients, drugs, and at the end of the day, it generates a report.

IR: Aaah... Okay, and is this system linked to all hospitals or is it only provided here at XXX?

RT: This is the WHO system; we use the WHO tool, but as a system, no, we expect that when we get our own machine, it will process the data and help us get reports. So currently, you enter each patient's data manually.

IR: What activities are conducted under the umbrella of your AMS team here?

RT: AMS, first, preparing that Antibiogram, secondly, we do surface culture, we have been interviewed by IPC team, we attend IPC meetings, and QI [quality improvement]. We also do sensitization; recently, I finished organizing a certain project about handwashing, to inform someone regarding how it relates to drug resistance, as you can get hospital infections that lead to a challenge of drug resistance and those pathogens can bring challenges.

IR: And regarding the guidelines for drug dispensing, pharmacists and doctors...

RT: Yeah, thank you... I had forgotten a bit. Also, in our action plan, there was supposed to be that policy which we assigned the secretary to do those things, to lead the pharmacists in drug dispensing.

IR: Are there national guidelines or are you using your own?

RT: So, we have also developed our own.

IR: Which is in the process, hasn't started yet...

RT: Yeah.

IR: And finally, in your facility, are there drugs that need authorization before being dispensed, and who authorizes them?

RT: Maybe following that national guidelines name list, I think there it is direct, and we treat according to the guidelines, so there are drugs without specialists that do not come out... until a specialist prescribes, and then we can provide the service.

IR: Okay... and perhaps you conduct Antibiotic reviews, I mean audit reports, by any means. Do you discuss them with doctors or just provide them with reports and they continue?

RT: We once did this equivalency survey project.

IR: Did you share feedback with them, like how it was received, what was the feedback? Did they receive it well, or did it just end with the report?

RT: Actually, in our structure, in that small committee with different people, we provided because we can't collect from the whole hospital, but there are members from different departments like Internal medicine, Surgery Department, each one has a representative, so we made a presentation to convey the message to them. But there were some challenges because as I said, doctors have some challenges.

IR: What challenges?

RT: They are not yet ready to change... they believe they are everything.

IR: What is the problem, or complaint, or something that prevents them?

RT: The belief that, for example, let's say they were questioning about going there often because we went there and found that some patients were given drugs, for example, cetirizine, the surgery team does not follow the required procedures, they are prophylaxis but you find another one has not followed the prophylaxes procedure, so if you correct them a bit, they have many questions, they say no, this causes this…. When you ask them if they feel they are left without evidence, so there is a challenge like that, or sometimes you find a child is vomiting, for example, you find they are given something else in the age were they are prone to Rotavirus, they gave the child a drug without testing, they give something else, so there are challenges, they give antibiotics, so the challenge is like that…

IR: And there are tests for Rotavirus and Adenovirus?

RT: Tests are there, not for Rotavirus but we can even do stool culture or stool analysis….

IR: So the big challenge is in receiving these feedbacks, that samples need to be tested first to get results…or maybe it could be pressure from patients. That patients pressure doctors to give them drugs? That they should not lose that patient.

IR: So the big issue is that the patient does not disappear, yet there is also a push to reduce mortality.

IR: Okay, and you said now it's the third year since you started these programs, what things contribute to the continuity of this program or facilitate this program despite these challenges here, and what are the facilitators that help the program to exist and continue working?

RT: First, I congratulate Chief MOI and his department, and then I congratulate the ministry, that push, the secretariat multisectoral committee that comes for supervision, so they identified that gap and said we are needed, so that push helped us to have that committee.

IR: So it's just supervision, there are no incentives?

RT: Maybe there are no incentives at all, so some members sometimes feel burdened.

IR: And you said that when you call it a team, you call people from various sections, so how many people are in XXX?

RT: In the team, as a team, we have about 13 people, but we select some members because we want to convey the message effectively.

IR: How many have you selected?

RT: AAH,,about seven, but not all of them are active in the team…only four are mostly active

IR: Aaah, any other challenges apart from those you mentioned about doctors maybe...

RT: Financial challenges... the AMS committee has no budget.

IR: No budget?

RT: Yes, and there are many activities, for example, today if someone wants to do a point prevalence survey, it's as research, for example to do a survey in children's ward, the children's ward alone, there are minimum of 80 patients or you just want to do prescription survey because all those are AMS activities, but you find when they look at it, they sees all that time and there is not even any payment, there is not even a small allowance that makes it difficult.

IR: And in terms of resources, maybe laboratory or pharmacy and the hospital in general?

RT: Resources are a challenge in one place, the availability of antibiotic discs.

IR: For susceptibility?

RT: Yes, because we in government hospitals depend on MSD, and you find that MSD doesn't have, so you find it a long process until you get those discs, which also leads to having fewer

drugs, you can't do good sensitivity, you can't prepare good Antibiogram, so that's the challenge we face.

IR: So if such an issue arises, do you select for each patient who requests to do culture?

RT: In the past when we were not having high culture number, but now days it has increased, it has increased because some patients from private come to government after the insurance puts limits in NHIF insurance.

IR: What are the limits?

RT: The number of patients here is slowly increasing now and we expect that as the number increases and the portion of the sample increases, we have seen that on the laboratory side, so we want to advance a little, at least to have machines like VITECH or BIOTECH, we are still in that process so that we do not have stockout of that disc.

IR: And has it ever happened that the discs have run out and the Antibiogram sensitivity test is not done?

RT: They can decrease but it is impossible to run out completely.

IR: It's impossible... alright.

RT: But in some cases, you can find that all the drugs have become resistant, and you don't have other options, you might want to move to other techniques but find that particular drug is not available.

IR: So you have to continue with the resources you have...?

RT: Yes, the resources you have.

IR: And what do you suggest could help support the implementation of these programs to perform well for doctors, for you as AMS team members, for patients, and for the entire program?

RT: I think we still need to first sensitize the doctors, either we create another policy that can restrict them more in prescribing drugs because if a patient comes and complains, the lab results say one thing but they still give antibiotics, so that brings challenges. They need to change and understand what the patient really needs, according to the results and their history. If a patient needs medication, it could just be stress; you tell the patient they have an infection, we end up providing feedback in the future, but we have the ability to confirm the infection thoroughly through culture, what caused the infection, what should we do, but mainly we treat empirically, so it's time to move on to change. Someone says it's easy, another stresses out, and they fail to do certain things; you give antibiotics to someone who shouldn't have them, or someone has a cold, and you give them antibiotics, so there are things like that. However, AMS itself lacks financial support because if you look at AMS, it has many activities that can overload staff in their allocated work time. First, they need extra time to do such work, so someone can't do that; they need stationery, they need materials to do such work, so the financial issue is also a challenge.

IR: Thank you very much for your time, but before we finish, maybe there's something else that you think is a valuable addition for AMS at XXX or other hospitals?

RT: Sensitization.

IR: (Laughs) It's still sensitization?

RT: It's a challenge that we're still facing...

IR: It's a big one.

RT: Because if we haven't done enough sensitization, people won't change, so every day we provide training, we sit and listen to the effects of resistance and also the management of patients, how we failed to utilize microbiology department.

IR: Thank you very much for your time, and I really appreciate the information you've provided.
