# Supplementary material for: Barriers and facilitators for the implementation of Antimicrobial Stewardship Programs in Dar es Salaam Regional Referral Hospitals (RRHs)
Source: PLOS Glob Public Health. 2026 Mar 23;6(3):e0006123. doi: 10.1371/journal.pgph.0006123 (PMC13008068; doi:10.1371/journal.pgph.0006123)
Supplement: S1 Data — (ZIP) [file pgph.0006123.s002.zip › Transcript_01_merged_compressed.pdf]

RESPONDENT (RT)

INTERVIEWER (IR)

IR: As I introduced myself earlier, my name is Bethania Magesa, a master's student at Muhimbili University, pursuing a master's degree in Monitoring and Evaluation in Health. I am currently conducting research to assess the effectiveness of Antimicrobial Stewardship (AMS) teams in reducing the burden of antimicrobial resistance in Dar es Salaam RRHs. I understand that our referral hospitals in Dar es Salaam have been capacitated to establish these AMS programs, and XXX, as one of the hospitals, benefits from these programs. So, I'd like to know who you are, perhaps your profession, whether you're a pharmacist, nurse, doctor, and your involvement in these AMS programs here at Temeke?

RT: Ah, okay, my name is XXX. I am a pharmacist and the secretary of the AMS team. Yes, and I am also the in-charge pharmacist of the inpatient pharmacy.

IR: Okay, from your understanding, what is AMS, or what are these programs?

RT: AMS programs primarily deal with how antimicrobials are used, considering the priorities set by the new AMS guidelines, which run from 2022 to 2027. Importantly, we focus on the right choice of antimicrobials for specific pathogens. But we also look at the proper use of these antimicrobials to avoid resistance. Proper use depends on how we perform or how medications are dispensed to our clients, considering the entire process, starting with good diagnostic stewardship. The doctor listens carefully, and if there are tests, they follow the same sequence. For example, if cultures are taken based on the patient's condition, the results are returned to the doctor, who then makes decisions based on the lab results. Currently, we emphasize using culture and sensitivity tests frequently and are developing an antibiogram to guide us on which medications to use for empirical treatment and specific types of infections. We are working on this. Previously, we could get isolates and perform cultures on about 160 to 200 patients annually, but now we can do 2000 to 3000 due to changes in equipment and emphasis on culture testing. We also train doctors to emphasize culture testing to get the best treatment options. You know, in most of our referral hospitals, when a patient arrives, they often use ceftriaxone, which has become a challenge because it is no longer effective. We decided to develop an antibiogram to determine which medications are resistant and which are the best options. We are also developing a hospital formulary. I was the one to write the hospital formulary, tailoring it to our environment and management practices to make it easier for doctors to know which cultures to perform for specific medications.

IR: I see. How do you implement these programs, and when did they start here at XXX?

RT: The programs started at XXX around 2021. We participated in training provided by the Ministry of Health, which guided us on how to deal with antimicrobial resistance and control infection prevention and control (IPC) to prevent hospital-acquired infections. Resistance is often due to how antiseptics are used for cleaning. If they are weak, they can cause infections and resistance to many medications. So, we dealt with IPC, which had good control measures.

IR: Ah, okay. Does your institution here at XXX receive any financial or budgetary assistance or support from the Ministry or implementing partners in terms of financial assistance or anything else?

RT: In terms of financial assistance and training, we have received support directly from the Ministry of Health, sponsored by MTaPS. However, there have been challenges, especially in conducting research. Sometimes, I do research on antibiotic use and write about it, but it's often at my own expense.

IR: At your own expense?

RT: Yes, so that becomes a challenge. But we have requested a budget in the new plan to conduct quarterly evaluations of resistance and other aspects. Hopefully, in the coming year, we will be better supported. Previously, we conducted quality equivalency studies and research about five times as a point of reference.

IR: And what is the trend?

RT: The trend varies by ward. There was overuse of ceftriaxone and penicillin, which are no longer effective. For example, in surgical wards, there was no guide for prophylaxis, so everyone did what they could based on their area, which led to many challenges.

IR: What is your perspective on the AMS programs at XXX in terms of improving or maintaining the quality of services, medications, and everything related to XXX?

RT: Firstly, we can say it's not just about XXX. It's about the patient. By reducing patient sterilization, we avoid many costs. For instance, we reduced the cost of culture tests from 20,000 Tanzanian shillings to 10,000, making it easier for patients to afford and for doctors to order tests. Initially, we conducted 160 to 200 cultures annually, but now we do 2500 to 3000.

IR: Annually?

RT: Yes, and we plan to acquire a machine that will speed up culture tests, reducing the time from five to seven days to three days, allowing for quicker patient management and discharge, thus reducing hospitalization costs and improving efficiency.

IR: Has there been any change in the delivery of antibiotics or services provided by healthcare providers since AMS was introduced at XXX? Has empirical treatments decreased or changed?

RT: Empirical treatment with ceftriaxone, metronidazole, and ampicillin remains a challenge. We are developing guidelines based on sample sizes of more than 20,000 to identify the best initial medications for specific conditions before performing cultures. We emphasize that patients with antimicrobial needs should have samples taken before starting treatment to ensure they receive the right medication and reduce resistance and unnecessary medication use. We expect to have a full picture by December and start arranging the guidelines.

IR: When did you start preparing the antibiogram, this year or last year?

RT: Last year. Previously, it was difficult due to a lack of necessary supplies for the lab, but we received supplies last year, enabling us to progress.

IR: So, we expect to have the important antibiogram this year?

RT: Yes.

IR: Do you have specific treatment guidelines for certain diseases developed here at XXX or what guidelines do you use?

RT: We use STG guidelines and are supported by the Hospital Formulary, which includes authorized medications specific to XXX. It's a comprehensive book with medication information.

IR: Are there any antibiotics that require preauthorization, and who is responsible for preauthorization?

RT: Most medications rely on specialists in that area, and preauthorization requires evidence-based justification. We don't do anything without evidence.

IR: And you use these pre-authorization studies as antibiotic reviews, right?

RT: Yes, and we present them to the therapeutic committee to get a full picture of our progress.

IR: Do you share feedback with other healthcare providers, and what feedback do you receive from them?

RT: We present to the therapeutic committee weekly, every Monday or Tuesday, where heads of all departments are present and informed about the progress.

IR: And how is their feedback to you?

RT: The feedback we rely on is their performance. When we see changes, we know they understand.

IR: Okay, we've discussed many things since AMS was established in 2021. Now it's 2024, and you've been on the team all this time. What do you think contributes to the success of the AMS team here at XXX, or what drives its continued existence for nearly four years?

RT: Ah, any success depends on leadership. With supportive leadership and active personnel, everything goes well. Initially, I didn't know I was part of AMS. But when writing the Hospital Formulary, I got many ideas, and eventually, I became the secretary this year.

IR: Good leadership has ensured the AMS program runs smoothly.

RT: Yes, it has.

IR: Okay. What challenges do healthcare providers or patients face, or what hinders the team from performing its duties as expected?

RT: One challenge is responsibilities. For example, AMS members are supposed to be on ward rounds every morning, but due to time constraints, we only manage to do it on Friday. Another challenge is funding. Nothing works without money. We need supplies for documentation and other things, and sometimes we work extra hours without reimbursement. We proposed including AMS duties in official letters to motivate team members.

IR: So, they were not refunded?

RT: Yes, that's why we proposed including AMS duties in official letters to provide extra motivation. Another challenge is recruiting and training interns to understand AMS, which can be difficult as they come from different backgrounds. We rely on IPC members, like the sister you saw, to assist.

IR: Ah, okay. Thank you very much for your time. Is there anything else you'd like to add about AMS at XXX?

RT: About XXX, there's not much more to add. We are thankful for the Ministry's inspections and the positive feedback we've received. The main challenges are the small issues I've mentioned and the fact that some key team members have been transferred, which reduces team strength and requires training new members.

IR: Transferred, not resigning?

RT: Yes, they requested transfers and were granted, so we lost some of the best team members. However, we continue to monitor and ensure proper medication use and cultures.

IR: Thank you very much for your time and input.

RESPONDENT (RT)

INTERVIEWER (IR)

IR: As I introduced myself earlier, my name is Berthania Magesa, a master's student at Muhimbili University, pursuing a master's degree in Monitoring and Evaluation in Health. I am currently conducting research to assess the effectiveness of Antimicrobial Stewardship (AMS) teams in reducing the burden of antimicrobial resistance in Dar es Salaam RRHs. I understand that our referral hospitals in Dar es Salaam have been capacitated to establish these AMS programs, and XXX, as one of the hospitals, benefits from these programs.

IR: So, I would like to start with the first question to understand which language you prefer to use.

RT: Swahili.

IR: You are allowed to mix if you wish... Alright, I would like to know your understanding about these Antimicrobial Stewardship programs and when they started at XXX?

RT: These programs at XXX have two aspects. There is a national indirect lab part where they bring QA [quality assurance] to look at resistance. But for our facility, we started following up about two years ago, and it has been effective since last year after we went for training and started the follow-up. Before that, about two or three years ago, I cannot say exactly how many years, but around two or three.

IR: And how are you involved in these programs, in other words, what role do you play in the AMS team?

RT: My role here at XXX, as I told you... first, I am the chairperson of AMS. Before being the chairperson, because I am on this side of the laboratory and I am the main producer of microbiology data, we were doing Antibiogram and also surface toucher to observe the resistance trend. So, to participate, I do it in two ways: as a chairperson, and at the same time, I am in the microbiology department.

IR: Does the institution receive any support in terms of training or allowances for those in the AMS team?

RT: Mhh, no allowances for the AMS team, but we received training and have been trained.

IR: And is there perhaps surveillance supervision?

RT: Surveillance, yes, from the ministry.

IR: How often does it happen? Quarterly?

RT: Let me say quarterly, I don't have the exact... it's quarterly.

IR: And what is your overall view regarding these programs since they started here?

RT: The AMS ones?

IR: Yes.

RT: Ahaaa, my view is still with the doctors because they are the main prescribers of drugs. Sometimes you know when people want to change, they take time. You can provide culture results but the patient has already received drugs.

IR: Okay, has it helped to improve the quality of prescriptions or has it helped to reduce the burden of Antimicrobial Resistance since they started, roughly?

RT: It has increased the burden.

IR: Increased the burden? Even though the programs exist, they are not implemented as required?

RT: Although for our laboratory side, we have a program we want to advance. We even want to invite some people to come for microbiology-related training. The department enabled us to do microbiology sensitization so that they are aware of AMS and such things.

IR: Do you provide reports on antibiotic resistance Antibiogram data?

RT: Now, Antibiogram, I told you we started about three years ago. In the period we were doing it, it wasn't up to standard; we were doing it quarterly, although the number of isolates didn't meet the standards. But we got training, we became more advanced and aware, and we are supposed to prepare Antibiogram. So currently, we are in the phase of collecting those isolates, which we started in November last year, and we are still using traditional methods and global reporting system.

IR: Is this a WHO system?

RT: It's the WHO microbiology system itself that includes patients and those isolates, patients, drugs, and at the end of the day, it generates a report.

IR: Aaah... Okay, and is this system linked to all hospitals or is it only provided here at XXX?

RT: This is the WHO system; we use the WHO tool, but as a system, no, we expect that when we get our own machine, it will process the data and help us get reports. So currently, you enter each patient's data manually.

IR: What activities are conducted under the umbrella of your AMS team here?

RT: AMS, first, preparing that Antibiogram, secondly, we do surface culture, we have been interviewed by IPC team, we attend IPC meetings, and QI [quality improvement]. We also do sensitization; recently, I finished organizing a certain project about handwashing, to inform someone regarding how it relates to drug resistance, as you can get hospital infections that lead to a challenge of drug resistance and those pathogens can bring challenges.

IR: And regarding the guidelines for drug dispensing, pharmacists and doctors...

RT: Yeah, thank you... I had forgotten a bit. Also, in our action plan, there was supposed to be that policy which we assigned the secretary to do those things, to lead the pharmacists in drug dispensing.

IR: Are there national guidelines or are you using your own?

RT: So, we have also developed our own.

IR: Which is in the process, hasn't started yet...

RT: Yeah.

IR: And finally, in your facility, are there drugs that need authorization before being dispensed, and who authorizes them?

RT: Maybe following that national guidelines name list, I think there it is direct, and we treat according to the guidelines, so there are drugs without specialists that do not come out... until a specialist prescribes, and then we can provide the service.

IR: Okay... and perhaps you conduct Antibiotic reviews, I mean audit reports, by any means. Do you discuss them with doctors or just provide them with reports and they continue?

RT: We once did this equivalency survey project.

IR: Did you share feedback with them, like how it was received, what was the feedback? Did they receive it well, or did it just end with the report?

RT: Actually, in our structure, in that small committee with different people, we provided because we can't collect from the whole hospital, but there are members from different departments like Internal medicine, Surgery Department, each one has a representative, so we made a presentation to convey the message to them. But there were some challenges because as I said, doctors have some challenges.

IR: What challenges?

RT: They are not yet ready to change... they believe they are everything.

IR: What is the problem, or complaint, or something that prevents them?

RT: The belief that, for example, let's say they were questioning about going there often because we went there and found that some patients were given drugs, for example, cetirizine, the surgery team does not follow the required procedures, they are prophylaxis but you find another one has not followed the prophylaxes procedure, so if you correct them a bit, they have many questions, they say no, this causes this…. When you ask them if they feel they are left without evidence, so there is a challenge like that, or sometimes you find a child is vomiting, for example, you find they are given something else in the age were they are prone to Rotavirus, they gave the child a drug without testing, they give something else, so there are challenges, they give antibiotics, so the challenge is like that…

IR: And there are tests for Rotavirus and Adenovirus?

RT: Tests are there, not for Rotavirus but we can even do stool culture or stool analysis….

IR: So the big challenge is in receiving these feedbacks, that samples need to be tested first to get results…or maybe it could be pressure from patients. That patients pressure doctors to give them drugs? That they should not lose that patient.

IR: So the big issue is that the patient does not disappear, yet there is also a push to reduce mortality.

IR: Okay, and you said now it's the third year since you started these programs, what things contribute to the continuity of this program or facilitate this program despite these challenges here, and what are the facilitators that help the program to exist and continue working?

RT: First, I congratulate Chief MOI and his department, and then I congratulate the ministry, that push, the secretariat multisectoral committee that comes for supervision, so they identified that gap and said we are needed, so that push helped us to have that committee.

IR: So it's just supervision, there are no incentives?

RT: Maybe there are no incentives at all, so some members sometimes feel burdened.

IR: And you said that when you call it a team, you call people from various sections, so how many people are in XXX?

RT: In the team, as a team, we have about 13 people, but we select some members because we want to convey the message effectively.

IR: How many have you selected?

RT: AAH,,about seven, but not all of them are active in the team…only four are mostly active

IR: Aaah, any other challenges apart from those you mentioned about doctors maybe...

RT: Financial challenges... the AMS committee has no budget.

IR: No budget?

RT: Yes, and there are many activities, for example, today if someone wants to do a point prevalence survey, it's as research, for example to do a survey in children's ward, the children's ward alone, there are minimum of 80 patients or you just want to do prescription survey because all those are AMS activities, but you find when they look at it, they sees all that time and there is not even any payment, there is not even a small allowance that makes it difficult.

IR: And in terms of resources, maybe laboratory or pharmacy and the hospital in general?

RT: Resources are a challenge in one place, the availability of antibiotic discs.

IR: For susceptibility?

RT: Yes, because we in government hospitals depend on MSD, and you find that MSD doesn't have, so you find it a long process until you get those discs, which also leads to having fewer

drugs, you can't do good sensitivity, you can't prepare good Antibiogram, so that's the challenge we face.

IR: So if such an issue arises, do you select for each patient who requests to do culture?

RT: In the past when we were not having high culture number, but now days it has increased, it has increased because some patients from private come to government after the insurance puts limits in NHIF insurance.

IR: What are the limits?

RT: The number of patients here is slowly increasing now and we expect that as the number increases and the portion of the sample increases, we have seen that on the laboratory side, so we want to advance a little, at least to have machines like VITECH or BIOTECH, we are still in that process so that we do not have stockout of that disc.

IR: And has it ever happened that the discs have run out and the Antibiogram sensitivity test is not done?

RT: They can decrease but it is impossible to run out completely.

IR: It's impossible... alright.

RT: But in some cases, you can find that all the drugs have become resistant, and you don't have other options, you might want to move to other techniques but find that particular drug is not available.

IR: So you have to continue with the resources you have...?

RT: Yes, the resources you have.

IR: And what do you suggest could help support the implementation of these programs to perform well for doctors, for you as AMS team members, for patients, and for the entire program?

RT: I think we still need to first sensitize the doctors, either we create another policy that can restrict them more in prescribing drugs because if a patient comes and complains, the lab results say one thing but they still give antibiotics, so that brings challenges. They need to change and understand what the patient really needs, according to the results and their history. If a patient needs medication, it could just be stress; you tell the patient they have an infection, we end up providing feedback in the future, but we have the ability to confirm the infection thoroughly through culture, what caused the infection, what should we do, but mainly we treat empirically, so it's time to move on to change. Someone says it's easy, another stresses out, and they fail to do certain things; you give antibiotics to someone who shouldn't have them, or someone has a cold, and you give them antibiotics, so there are things like that. However, AMS itself lacks financial support because if you look at AMS, it has many activities that can overload staff in their allocated work time. First, they need extra time to do such work, so someone can't do that; they need stationery, they need materials to do such work, so the financial issue is also a challenge.

IR: Thank you very much for your time, but before we finish, maybe there's something else that you think is a valuable addition for AMS at XXX or other hospitals?

RT: Sensitization.

IR: (Laughs) It's still sensitization?

RT: It's a challenge that we're still facing...

IR: It's a big one.

RT: Because if we haven't done enough sensitization, people won't change, so every day we provide training, we sit and listen to the effects of resistance and also the management of patients, how we failed to utilize microbiology department.

IR: Thank you very much for your time, and I really appreciate the information you've provided.

IR: Interviewer

RT: Respondent

IR: As I introduced myself earlier, my name is Berthania Paul Magesa, a second-year student pursuing a master's degree in project management and Evaluation at Muhimbili University of Health and Allied Sciences. I am conducting research on the effectiveness of Antimicrobial Stewardship Programs to reduce drug resistance in referral hospitals in the Dar es Salaam region. Ah, I'm not sure which language you prefer to use?

R: Ah, I'll use Swahili.

IR: And what is your profession?

RT: I am a pharmacist.

IR: Ah, what do you understand by the term Antimicrobial Stewardship Programs?

RT: Okay, as I understand it, it's generally about plans or strategies that enable the appropriate use of antibiotics or antimicrobial agents in general.

IR: Who is supposed to be involved in AMS?

RT: Those who should be involved are all AMS workers, but also our clients, meaning all patients should be involved.

IR: Okay, understood. And how long have these programs been in place at this hospital?

RT: Hmm, since they were established, they became effective last year. They started becoming effective last year, and that's when we formed a team. But before that, we were just participating without a team. This started back in the year two thousand and twenty-two.

IR: What activities are carried out under the umbrella of the AMS program or AMS Team here at XXX?

RT: First, we are involved, and because it also depends on the profession we belong to... because as a team, the people we oversee, we educate them for what purpose... for the first environment in the pharmacy department where those who sit in the dispensing unit ensure that they review those drug prescriptions to be dispensed accurately. But another thing, as the AMS team, we also visit the wards to check how antibiotics prescriptions are being dispensed, and we provide recommendations. Yeah... if there is a drug showing resistance and such things, then we provide recommendations to reduce it. But another thing, in the laboratory department, they help us to provide those Antibiograms which assist the doctor in empirical treatment in case the culture results are delayed, so that Antibiogram helps us in empirical treatment. So, these things are done partly in the laboratory.

IR: And what is your role within the AMS team here at XXX? What role do you play?

RT: Okay... In this AMS team at XXX, I am the team secretary first. As the team secretary, my role is to ensure that all AMS plans that are implemented are organized. I organize meetings that we need to have

at least once a month, although depending on responsibilities, sometimes we skip, but we try our best. That's the first thing. Also, I prepare the meeting agendas and generally participate in the assigned tasks.

IR: Ah, does the AMS team here at XXX receive any kind of financial support or assistance in terms of resources, supervision?

RT: Here at XXX, there are still challenges; we haven't received any yet, but it's in the plan because in these initial stages, as I mentioned earlier, it's something new. If it's something new, it means that in the hospital plans, the work plan of the hospital is not yet in place. So, because the work plan is not currently available, we don't have anything; it's like we are volunteering. But in the next phase, I think the hospital will be involved; we will get anything; there will be that support from the management, but for now, nothing yet.

IR: What is your view on the AMS feedback since it started? How have they been received at XXX by pharmacists, doctors, nurses, and other healthcare workers?

RT: The reception... initially, the reception was a bit difficult because they believed AMS was for pharmacists only. So, if nurses knew, "This is for pharmacists, medicine things," doctors would say, "Ah, medicine things are for pharmacists." So, they received it differently, but after those training sessions involving different people, which were also conducted by the ministry but also supervised by the ministry, they helped build the capacity of various cadres to understand that this issue is for everyone in the hospital to ensure the proper use of medicines and to reduce the misuse of medicines.

IR: And regarding the quality of services provided, have there been improvements since the AMS team started, maybe a reduction in empirical treatments?

RT: Yeah, I can say it has helped a lot in terms of quality. I can say it has helped because starting from the prescription, from how the prescriber prescribes the medicine, we don't have those errors anymore; they are very few compared to before. Right now, people follow those standard treatment guidelines because we as a team emphasize it. So, those errors in medicine have greatly reduced, yeah, they have reduced significantly.

IR: What were the errors before, and what are they now?

RT: Ahh, before, there was non-compliance with guidelines, for example, the STGs (Standard Treatment Guidelines) were not followed, so if you don't follow that, it's an error; it means you will prescribe the wrong dose and such things, and medicines and doses that are not supposed to be prescribed. So now, these things have greatly reduced automatically, and now, many prescriptions are correct.

IR: Okay, and how long does the hospital generate the susceptibility report?

RT: Ah, okay. Are you talking about the Antibiogram?

IR: Yes.

RT: Before, we used to produce them monthly, but after conducting training last year, a training we did with the ministry, the ministry taught us to produce these at least annually. Because annually, we will have enough data and enough bacteria, so it is appropriate to produce a complete Antibiogram. So right now, we are in the process of preparing a complete annual report, but previously, we used to prepare

them monthly, which also helped us, but now we want to prepare the annual one that we will use for a longer time.

IR: Okay. Before, you never produced an annual report, you used to produce monthly reports?

RT: Yeah, monthly reports, we used to produce them every month.

IR: Yeah, how were those reports received, were there any resistance in the reports?

RT: Ahh, okay. The resistance was normal, yeah, it was just some drugs that were fully resistant, and these were drugs that are also commonly used outside, like Ceftriaxone, most of the time, we had that problem, and drugs like Cipro didn't help. These are drugs that sometimes are very common outside.

IR: And you mentioned the standard treatment guidelines; does the hospital have its own guidelines for prescriptions and treatments that have been developed and disseminated to service providers?

RT: Okay, yeah, of course, we have a hospital formulary now that we got in December last year, that's when we printed it, so now we have a complete book. Before, for example, in 2022, we had a book that was in softcopy form, but now we have printed it, so we have it.

IR: And was it disseminated to everyone?

RT: Not everyone, but at least each unit has received it.

IR: And the issue of pre-authorization of antibiotics, who is responsible for which drugs often require pre-authorization for patients?

RT: Ah, okay. Here we have a challenge, especially for cash patients, but for insured patients, there is a specific procedure. Insured patients have more control because of the fear of deductions, where there is a special room number 44 upstairs, those people... although they use it, it's not just antibiotics; it means all drugs. It means drugs that are looked at very carefully because if you make a mistake, it becomes an error. So, there is a special room where they cross-check the drug before it is submitted to the NHIF; they review if the drug was dispensed correctly, everything. Cash patients remain with pharmacists because they are the last stage; they go and use it; they cross-check if there is accuracy, if there is a challenge, then the doctor will be given feedback to make adjustments to that prescription, yeah.

IR: Ah, what about inspecting antibiotics? Does the hospital do that?

RT: Yeah... the first thing is done by pharmacists, but even the team does it. We inspect the dispensing rooms of pharmacists; we inspect prescriptions to see which ones had errors, we look at which ones were correct...

IR: And feedback is given to service providers after reviews; how is the feedback mechanism between those who do these reviews and other service providers?

RT: The feedback is not very good; we had that plan, but...there are some challenges in receiving the feedback.

IR: What are the things that facilitate the implementation of ASP in XXX?

RT: We usually do CME meetings; if there is something we have seen that is hot and needs quick corrections, we usually present it at our CME meetings, which mostly reach many people.

IR: Aahh..okay, any other factors?

RT: First, the ministry has helped us a lot; it has helped us with training and has taught us, but also, supervision has helped us a lot by keeping us in line.

IR: And for how long was the supervision done?

RT: In terms of time, I may not be very accurate, but they used to come, so maybe twice a year, they used to come, such training as last year, we did it twice, because they combine IPC and AMS like twice in those trainings supervision comes, two or three times a year.

IR: What are the challenges that have occurred since AMS started until now?

RT: The first challenge... because the big thing is that we are in the hospital to save the patient's life, so we are busy treating the patient, but some of these things we sometimes forgotten, and even as a team, we sometimes don't do our duties because of each department, so it is difficult to connect us together. I think that's the biggest thing that has troubled us, that we are busy with our work areas, and the implementation of this team has been difficult for us.

IR: Are there any individual factors?

RT: Ahh, okay, for individuals, no, we haven't encountered those much because these things have decreased because in the past or a while ago, there were some, but you find that if you correct a doctor a doctor, he tells you that the medicine I wrote is the same, but now everything is guided, and easy to criticize yourself, so broadly, we have reduced those barriers individually.

IR: Ah, okay, thank you very much for your time to participate in this interview. Is there any additional point you would like to add?

RT: Ah, thank you very much; this is really good. I would advise you to do it well; we expect to see your publications helping because that's the problem now; people misuse medicines, random use. I mean, now this is what leads to these antibiotics being misused, and later, we may lack medicines for treatment, so these publications will help us and the community also know about the use of these antibiotics. I'm grateful; it's a good topic you've chosen.

IR: Thank you very much for your cooperation.

RT: Okay.

IR: Interviewer

RT: Respondent

IR: As I introduced myself, my name is Berthania Paul Magesa, I am a second-year master's student at the Muhimbili University of Health and Allied Sciences. I am studying Monitoring and Evaluation in Health and currently conducting research on implementation efficiency of AMS programs in reducing the magnitude of antibiotic resistance in referral hospitals in Dar es Salaam. Could you briefly tell me, what is your role and how do you participate in the AMS team here in XXX?

RT: Ahhh, my name is YYY, I am the lab scientist at XXX Regional Referral Hospital. I am a member of the AMS champion team, meaning that the laboratory is the hub for testing antibiotic resistance caused by various drugs.

IR: Alright, and how long has AMS been established here at XXX?

RT: This is XXX...

IR: I mean XXX, sorry...

RT: AMS started immediately after the launch of the AMR programs. XXX is one of the AMR sight centers, including XXX, Morogoro, Maweni, and Benjamin Mkapa. Before that, we started with AMR in 2021, but we began actively participating in AMS in 2022.

IR: Ahh, what activities are carried out under the AMS umbrella?

RT: Ah, the activities that are carried out...

IR: In terms of any activities...?

RT: Ah, okay. The activities conducted here include laboratory work, primarily focusing on culture and sensitivity. Our main task is culture and sensitivity testing, but we also provide advice on the appropriate use of antibiotics before starting treatment.

IR: And overall, what activities are carried out in the hospital by the AMS team? What roles or tasks are performed?

RT: Ah, our main task is more than just testing; we also test patient samples.

IR: Yes.

RT: Where we get results and we do sensitivity tests to know if either it is sensitive or it is resistance. But we also do environmental sterility check we do swabbing in different environments like the theater, mortuary, other laboratories, and many places that we think there might be organisms to check for sterility in the environment. But we also provide advice to other users of laboratories. Hospitals in general regarding the results we get from culture and sensitivity, including this Antibiogram that we have developed, we share with clinicians to know what drugs may be suitable for our hospital setting, except for that of National wise.

IR: And does this AMS team receive any financial assistance, training, or supportive supervision from any ministry or community partners?

RT: Ah, we received training and supervision. The trainings we received were in-house, which was a mode of supervision, and these were mostly facilitated by MTaps. MTaps have been conducting supervision in collaboration with the ministry, I believe. So, the main support we have received is in terms of training and supportive supervision from MTaps.

IR: And what is your view on all these AMS programs? Do they help reduce antibiotic resistance here in XXX and have they helped improve the quality of services provided? What is your perspective on these teams since they started here in XXX up to now and how they operate?

RT: My perspective is that I have seen that it is clear that they have first raised awareness of resistance, which was there. You know, there may be resistance, but people were not aware of it because it was not talked about. So, in practice, the collaboration between AMS and AMR has raised awareness that perhaps was not previously thought about. You know, when you come to give feedback that a certain drug has a resistance of a certain level, people are surprised because it is a drug that they may have been prescribing extensively. So firstly, this has helped raise awareness, but it has also increased understanding even of the categories of drugs used, such as what is reserve and what is access, etc., which have been raised and discussed by AMS and AMR.

IR: And speaking on the side of producing Antibiogram data, have you produced any since AMS started?

RT: Yes, there is an Antibiogram that we have produced. Firstly, one that can be used and has met the criteria is from the year 2022….and the whole year, 2023. Before that, we produced one from 2021 to 2022, almost two years, but it did not meet the criteria because there were a number of organisms involved in this Antibiogram that were few. So we were told, for learning, we should develop Antibiogram but for us to be able to use it, we were told to increase the data. So we did more until 2023. We developed an Antibiogram that met the criteria.

IR: And has it been shared with other health workers?

RT: It has been shared with clinicians and I think it has been incorporated into the hospital's antibiotic drug guideline.

IR: And how was their perception? How was it received and what feedback did you get after providing this Antibiogram data?

RT: Ah, it was well received because, for example, when you talk about a drug, as I mentioned earlier, you talk about the resistance of the drug, which they clinicians thought was the best. The projected resistance pattern shows that there is a larger resistance pattern than the drug they prescribed. So, it was a good opportunity for them to change direction and to say this drug is not to be prescribed continuously because its resistance pattern has become larger. So it was well received.

IR: And from the time these programs started until now, what has facilitated these programs to continue working? Because here you said you produce Antibiogram data. What facilitates AMS teams to do their work and implement their responsibilities as required?

RT: Basically, what has facilitated AMS the most is the knowledge of how to do AMS. They have facilitated us a lot, but to get Antibiogram, AMR program, there was another AMR program that was supported by IDDS.

IR: Ah, IDDS are implementing partners?

RT: Yes, it was some sort of research. They had research for about five years, which started in 2020 and ended in March 2024. So, they supported even the material for doing culture, so it enabled us to develop the Antibiogram because to make that Antibiogram you must have a wide range of antibiotics involved in it. So, in normal circumstances, The utilization of microbiology department was somehow low, it was not given much priority before, unlike other sections like hematology, hematology, almost every hospital you find it in good condition but microbiology almost every hospital has little data but through AMR and AMS they have helped us to get almost all consumables basically they have built good capacity for us.

IR: And what challenges do personnel face in carrying out AMS responsibilities, both AMS members and other healthcare providers?

RT: Ah, there are challenges, although they have reduced somewhat. One challenge is that patients themselves find it difficult to wait for the results, you know, the Turnaround Time (TAT) for culture and sensitivity is a bit longer than other tests, so patients sometimes feel it's difficult to wait. As a result, doctors feel compelled to start treatment before receiving the results, which is called empirical treatment.

RT: Treatment based on... So if a patient shows improvement with empirical treatment, they might not return thinking they are cured, but it's just a temporary relief because the antibiotic has temporarily alleviated symptoms while the organism remains resistant and continues to grow. So, the first challenge

is the long TAT we have, Eeeh…. the second challenge we have is that the TAT remains high because we use conventional methods that are manual, so we do everything manually, so we don't have automated systems so far, so that's the challenge and the fact that we don't have automated systems TAT remains high…so I think long TAT also contributes to resistance.

[Both laugh]

RT: You see... so that challenge still exists, but we are trying to solve it, we have ordered automated although through the experience of our colleagues who have already received the automated there is still a challenge of operation...the cost of automated is very expensive and looking at the reality we have to increase the cost of culture and sensitivity to Tsh 20,000 /= , in the beginning we were doing at a cost of 10,000 / = only there we were supported by IDDS so IDDS have finished their program we automatically have to go back to the cost of reality we cannot continue with ten thousand you see…? Those are the challenges

IR: And for AMS team members, what challenges are there for healthcare providers or other healthcare providers?

RT: Aaah, other healthcare providers are not so much of a challenge AMS members for us here are staff turnover, for example we had a microbiologist here XXX has moved to Muhimbili so we don't have a microbiologist or a specialist in microbiology, but we have a specialized lab but it is insufficient.

IR: How insufficient?

RT: In terms of consumables and equipment, sometimes we have insufficient consumables to run microbiology section.

IR: What about the feedback from your fellow healthcare workers?

RT: Information is well received; it is not a challenge you know someone can receive information but fail to abide by it or don't want to be told the truth…..and patients are very many who want to be treated and go back home, many want to finish treatment on the same day…,so the issue of being told to return tomorrow to take the results is a bit of a challenge.

IR: Thank you very much for your time, maybe if there is anything else you would like to add before we finish these interviews recommendation or anything

RT: Eeeh…other recommendation is we had a big problem with these microbiology consumables from the medical stores department, many sensitivity disks were not in the system, you can find three or four disks in the system and the rest you have to ask for a special permit to procure…., so, you see that becomes a long chain..because you have to write a letter to MSD and this causes delay in getting the consumables…and this was because these disks were not used so much and MSD wants consumables that are ordered frequently, those which are not frequently ordered are frequently not in their warehouse….

IR: So should MSD improve their ordering system for microbiology consumables?

RT: Yes, because now microbiology labs are doing a lot of work in hospitals.

IR: Okay, Thank you very much for your time and input.

RT: Okay
