# Supplementary material for: Barriers and facilitators for the implementation of Antimicrobial Stewardship Programs in Dar es Salaam Regional Referral Hospitals (RRHs)
Source: PLOS Glob Public Health. 2026 Mar 23;6(3):e0006123. doi: 10.1371/journal.pgph.0006123 (PMC13008068; doi:10.1371/journal.pgph.0006123)
Supplement: S2 Data — (ZIP) [file pgph.0006123.s003.zip › Transcript_3_compressed.pdf]

IR: Interviewer

RT: Respondent

IR: As I introduced myself earlier, my name is Berthania Paul Magesa, a second-year student pursuing a master's degree in project management and Evaluation at Muhimbili University of Health and Allied Sciences. I am conducting research on the effectiveness of Antimicrobial Stewardship Programs to reduce drug resistance in referral hospitals in the Dar es Salaam region. Ah, I'm not sure which language you prefer to use?

R: Ah, I'll use Swahili.

IR: And what is your profession?

RT: I am a pharmacist.

IR: Ah, what do you understand by the term Antimicrobial Stewardship Programs?

RT: Okay, as I understand it, it's generally about plans or strategies that enable the appropriate use of antibiotics or antimicrobial agents in general.

IR: Who is supposed to be involved in AMS?

RT: Those who should be involved are all AMS workers, but also our clients, meaning all patients should be involved.

IR: Okay, understood. And how long have these programs been in place at this hospital?

RT: Hmm, since they were established, they became effective last year. They started becoming effective last year, and that's when we formed a team. But before that, we were just participating without a team. This started back in the year two thousand and twenty-two.

IR: What activities are carried out under the umbrella of the AMS program or AMS Team here at XXX?

RT: First, we are involved, and because it also depends on the profession we belong to... because as a team, the people we oversee, we educate them for what purpose... for the first environment in the pharmacy department where those who sit in the dispensing unit ensure that they review those drug prescriptions to be dispensed accurately. But another thing, as the AMS team, we also visit the wards to check how antibiotics prescriptions are being dispensed, and we provide recommendations. Yeah... if there is a drug showing resistance and such things, then we provide recommendations to reduce it. But another thing, in the laboratory department, they help us to provide those Antibiograms which assist the doctor in empirical treatment in case the culture results are delayed, so that Antibiogram helps us in empirical treatment. So, these things are done partly in the laboratory.

IR: And what is your role within the AMS team here at XXX? What role do you play?

RT: Okay... In this AMS team at XXX, I am the team secretary first. As the team secretary, my role is to ensure that all AMS plans that are implemented are organized. I organize meetings that we need to have

at least once a month, although depending on responsibilities, sometimes we skip, but we try our best. That's the first thing. Also, I prepare the meeting agendas and generally participate in the assigned tasks.

IR: Ah, does the AMS team here at XXX receive any kind of financial support or assistance in terms of resources, supervision?

RT: Here at XXX, there are still challenges; we haven't received any yet, but it's in the plan because in these initial stages, as I mentioned earlier, it's something new. If it's something new, it means that in the hospital plans, the work plan of the hospital is not yet in place. So, because the work plan is not currently available, we don't have anything; it's like we are volunteering. But in the next phase, I think the hospital will be involved; we will get anything; there will be that support from the management, but for now, nothing yet.

IR: What is your view on the AMS feedback since it started? How have they been received at XXX by pharmacists, doctors, nurses, and other healthcare workers?

RT: The reception... initially, the reception was a bit difficult because they believed AMS was for pharmacists only. So, if nurses knew, "This is for pharmacists, medicine things," doctors would say, "Ah, medicine things are for pharmacists." So, they received it differently, but after those training sessions involving different people, which were also conducted by the ministry but also supervised by the ministry, they helped build the capacity of various cadres to understand that this issue is for everyone in the hospital to ensure the proper use of medicines and to reduce the misuse of medicines.

IR: And regarding the quality of services provided, have there been improvements since the AMS team started, maybe a reduction in empirical treatments?

RT: Yeah, I can say it has helped a lot in terms of quality. I can say it has helped because starting from the prescription, from how the prescriber prescribes the medicine, we don't have those errors anymore; they are very few compared to before. Right now, people follow those standard treatment guidelines because we as a team emphasize it. So, those errors in medicine have greatly reduced, yeah, they have reduced significantly.

IR: What were the errors before, and what are they now?

RT: Ahh, before, there was non-compliance with guidelines, for example, the STGs (Standard Treatment Guidelines) were not followed, so if you don't follow that, it's an error; it means you will prescribe the wrong dose and such things, and medicines and doses that are not supposed to be prescribed. So now, these things have greatly reduced automatically, and now, many prescriptions are correct.

IR: Okay, and how long does the hospital generate the susceptibility report?

RT: Ah, okay. Are you talking about the Antibiogram?

IR: Yes.

RT: Before, we used to produce them monthly, but after conducting training last year, a training we did with the ministry, the ministry taught us to produce these at least annually. Because annually, we will have enough data and enough bacteria, so it is appropriate to produce a complete Antibiogram. So right now, we are in the process of preparing a complete annual report, but previously, we used to prepare

them monthly, which also helped us, but now we want to prepare the annual one that we will use for a longer time.

IR: Okay. Before, you never produced an annual report, you used to produce monthly reports?

RT: Yeah, monthly reports, we used to produce them every month.

IR: Yeah, how were those reports received, were there any resistance in the reports?

RT: Ahh, okay. The resistance was normal, yeah, it was just some drugs that were fully resistant, and these were drugs that are also commonly used outside, like Ceftriaxone, most of the time, we had that problem, and drugs like Cipro didn't help. These are drugs that sometimes are very common outside.

IR: And you mentioned the standard treatment guidelines; does the hospital have its own guidelines for prescriptions and treatments that have been developed and disseminated to service providers?

RT: Okay, yeah, of course, we have a hospital formulary now that we got in December last year, that's when we printed it, so now we have a complete book. Before, for example, in 2022, we had a book that was in softcopy form, but now we have printed it, so we have it.

IR: And was it disseminated to everyone?

RT: Not everyone, but at least each unit has received it.

IR: And the issue of pre-authorization of antibiotics, who is responsible for which drugs often require pre-authorization for patients?

RT: Ah, okay. Here we have a challenge, especially for cash patients, but for insured patients, there is a specific procedure. Insured patients have more control because of the fear of deductions, where there is a special room number 44 upstairs, those people... although they use it, it's not just antibiotics; it means all drugs. It means drugs that are looked at very carefully because if you make a mistake, it becomes an error. So, there is a special room where they cross-check the drug before it is submitted to the NHIF; they review if the drug was dispensed correctly, everything. Cash patients remain with pharmacists because they are the last stage; they go and use it; they cross-check if there is accuracy, if there is a challenge, then the doctor will be given feedback to make adjustments to that prescription, yeah.

IR: Ah, what about inspecting antibiotics? Does the hospital do that?

RT: Yeah... the first thing is done by pharmacists, but even the team does it. We inspect the dispensing rooms of pharmacists; we inspect prescriptions to see which ones had errors, we look at which ones were correct...

IR: And feedback is given to service providers after reviews; how is the feedback mechanism between those who do these reviews and other service providers?

RT: The feedback is not very good; we had that plan, but...there are some challenges in receiving the feedback.

IR: What are the things that facilitate the implementation of ASP in XXX?

RT: We usually do CME meetings; if there is something we have seen that is hot and needs quick corrections, we usually present it at our CME meetings, which mostly reach many people.

IR: Aahh..okay, any other factors?

RT: First, the ministry has helped us a lot; it has helped us with training and has taught us, but also, supervision has helped us a lot by keeping us in line.

IR: And for how long was the supervision done?

RT: In terms of time, I may not be very accurate, but they used to come, so maybe twice a year, they used to come, such training as last year, we did it twice, because they combine IPC and AMS like twice in those trainings supervision comes, two or three times a year.

IR: What are the challenges that have occurred since AMS started until now?

RT: The first challenge... because the big thing is that we are in the hospital to save the patient's life, so we are busy treating the patient, but some of these things we sometimes forgotten, and even as a team, we sometimes don't do our duties because of each department, so it is difficult to connect us together. I think that's the biggest thing that has troubled us, that we are busy with our work areas, and the implementation of this team has been difficult for us.

IR: Are there any individual factors?

RT: Ahh, okay, for individuals, no, we haven't encountered those much because these things have decreased because in the past or a while ago, there were some, but you find that if you correct a doctor a doctor, he tells you that the medicine I wrote is the same, but now everything is guided, and easy to criticize yourself, so broadly, we have reduced those barriers individually.

IR: Ah, okay, thank you very much for your time to participate in this interview. Is there any additional point you would like to add?

RT: Ah, thank you very much; this is really good. I would advise you to do it well; we expect to see your publications helping because that's the problem now; people misuse medicines, random use. I mean, now this is what leads to these antibiotics being misused, and later, we may lack medicines for treatment, so these publications will help us and the community also know about the use of these antibiotics. I'm grateful; it's a good topic you've chosen.

IR: Thank you very much for your cooperation.

RT: Okay.
