# Supplementary material for: Barriers and facilitators for the implementation of Antimicrobial Stewardship Programs in Dar es Salaam Regional Referral Hospitals (RRHs)
Source: PLOS Glob Public Health. 2026 Mar 23;6(3):e0006123. doi: 10.1371/journal.pgph.0006123 (PMC13008068; doi:10.1371/journal.pgph.0006123)
Supplement: S2 Data — (ZIP) [file pgph.0006123.s003.zip › Transcript_4_compressed.pdf]

IR: Interviewer

RT: Respondent

IR: As I introduced myself, my name is Berthania Paul Magesa, I am a second-year master's student at the Muhimbili University of Health and Allied Sciences. I am studying Monitoring and Evaluation in Health and currently conducting research on implementation efficiency of AMS programs in reducing the magnitude of antibiotic resistance in referral hospitals in Dar es Salaam. Could you briefly tell me, what is your role and how do you participate in the AMS team here in XXX?

RT: Ahhh, my name is YYY, I am the lab scientist at XXX Regional Referral Hospital. I am a member of the AMS champion team, meaning that the laboratory is the hub for testing antibiotic resistance caused by various drugs.

IR: Alright, and how long has AMS been established here at XXX?

RT: This is XXX...

IR: I mean XXX, sorry...

RT: AMS started immediately after the launch of the AMR programs. XXX is one of the AMR sight centers, including XXX, Morogoro, Maweni, and Benjamin Mkapa. Before that, we started with AMR in 2021, but we began actively participating in AMS in 2022.

IR: Ahh, what activities are carried out under the AMS umbrella?

RT: Ah, the activities that are carried out...

IR: In terms of any activities...?

RT: Ah, okay. The activities conducted here include laboratory work, primarily focusing on culture and sensitivity. Our main task is culture and sensitivity testing, but we also provide advice on the appropriate use of antibiotics before starting treatment.

IR: And overall, what activities are carried out in the hospital by the AMS team? What roles or tasks are performed?

RT: Ah, our main task is more than just testing; we also test patient samples.

IR: Yes.

RT: Where we get results and we do sensitivity tests to know if either it is sensitive or it is resistance. But we also do environmental sterility check we do swabbing in different environments like the theater, mortuary, other laboratories, and many places that we think there might be organisms to check for sterility in the environment. But we also provide advice to other users of laboratories. Hospitals in general regarding the results we get from culture and sensitivity, including this Antibiogram that we have developed, we share with clinicians to know what drugs may be suitable for our hospital setting, except for that of National wise.

IR: And does this AMS team receive any financial assistance, training, or supportive supervision from any ministry or community partners?

RT: Ah, we received training and supervision. The trainings we received were in-house, which was a mode of supervision, and these were mostly facilitated by MTaps. MTaps have been conducting supervision in collaboration with the ministry, I believe. So, the main support we have received is in terms of training and supportive supervision from MTaps.

IR: And what is your view on all these AMS programs? Do they help reduce antibiotic resistance here in XXX and have they helped improve the quality of services provided? What is your perspective on these teams since they started here in XXX up to now and how they operate?

RT: My perspective is that I have seen that it is clear that they have first raised awareness of resistance, which was there. You know, there may be resistance, but people were not aware of it because it was not talked about. So, in practice, the collaboration between AMS and AMR has raised awareness that perhaps was not previously thought about. You know, when you come to give feedback that a certain drug has a resistance of a certain level, people are surprised because it is a drug that they may have been prescribing extensively. So firstly, this has helped raise awareness, but it has also increased understanding even of the categories of drugs used, such as what is reserve and what is access, etc., which have been raised and discussed by AMS and AMR.

IR: And speaking on the side of producing Antibiogram data, have you produced any since AMS started?

RT: Yes, there is an Antibiogram that we have produced. Firstly, one that can be used and has met the criteria is from the year 2022….and the whole year, 2023. Before that, we produced one from 2021 to 2022, almost two years, but it did not meet the criteria because there were a number of organisms involved in this Antibiogram that were few. So we were told, for learning, we should develop Antibiogram but for us to be able to use it, we were told to increase the data. So we did more until 2023. We developed an Antibiogram that met the criteria.

IR: And has it been shared with other health workers?

RT: It has been shared with clinicians and I think it has been incorporated into the hospital's antibiotic drug guideline.

IR: And how was their perception? How was it received and what feedback did you get after providing this Antibiogram data?

RT: Ah, it was well received because, for example, when you talk about a drug, as I mentioned earlier, you talk about the resistance of the drug, which they clinicians thought was the best. The projected resistance pattern shows that there is a larger resistance pattern than the drug they prescribed. So, it was a good opportunity for them to change direction and to say this drug is not to be prescribed continuously because its resistance pattern has become larger. So it was well received.

IR: And from the time these programs started until now, what has facilitated these programs to continue working? Because here you said you produce Antibiogram data. What facilitates AMS teams to do their work and implement their responsibilities as required?

RT: Basically, what has facilitated AMS the most is the knowledge of how to do AMS. They have facilitated us a lot, but to get Antibiogram, AMR program, there was another AMR program that was supported by IDDS.

IR: Ah, IDDS are implementing partners?

RT: Yes, it was some sort of research. They had research for about five years, which started in 2020 and ended in March 2024. So, they supported even the material for doing culture, so it enabled us to develop the Antibiogram because to make that Antibiogram you must have a wide range of antibiotics involved in it. So, in normal circumstances, The utilization of microbiology department was somehow low, it was not given much priority before, unlike other sections like hematology, hematology, almost every hospital you find it in good condition but microbiology almost every hospital has little data but through AMR and AMS they have helped us to get almost all consumables basically they have built good capacity for us.

IR: And what challenges do personnel face in carrying out AMS responsibilities, both AMS members and other healthcare providers?

RT: Ah, there are challenges, although they have reduced somewhat. One challenge is that patients themselves find it difficult to wait for the results, you know, the Turnaround Time (TAT) for culture and sensitivity is a bit longer than other tests, so patients sometimes feel it's difficult to wait. As a result, doctors feel compelled to start treatment before receiving the results, which is called empirical treatment.

RT: Treatment based on... So if a patient shows improvement with empirical treatment, they might not return thinking they are cured, but it's just a temporary relief because the antibiotic has temporarily alleviated symptoms while the organism remains resistant and continues to grow. So, the first challenge

is the long TAT we have, Eeeh…. the second challenge we have is that the TAT remains high because we use conventional methods that are manual, so we do everything manually, so we don't have automated systems so far, so that's the challenge and the fact that we don't have automated systems TAT remains high…so I think long TAT also contributes to resistance.

[Both laugh]

RT: You see... so that challenge still exists, but we are trying to solve it, we have ordered automated although through the experience of our colleagues who have already received the automated there is still a challenge of operation...the cost of automated is very expensive and looking at the reality we have to increase the cost of culture and sensitivity to Tsh 20,000 /= , in the beginning we were doing at a cost of 10,000 / = only there we were supported by IDDS so IDDS have finished their program we automatically have to go back to the cost of reality we cannot continue with ten thousand you see…? Those are the challenges

IR: And for AMS team members, what challenges are there for healthcare providers or other healthcare providers?

RT: Aaah, other healthcare providers are not so much of a challenge AMS members for us here are staff turnover, for example we had a microbiologist here XXX has moved to Muhimbili so we don't have a microbiologist or a specialist in microbiology, but we have a specialized lab but it is insufficient.

IR: How insufficient?

RT: In terms of consumables and equipment, sometimes we have insufficient consumables to run microbiology section.

IR: What about the feedback from your fellow healthcare workers?

RT: Information is well received; it is not a challenge you know someone can receive information but fail to abide by it or don’t want to be told the truth…..and patients are very many who want to be treated and go back home, many want to finish treatment on the same day…,so the issue of being told to return tomorrow to take the results is a bit of a challenge.

IR: Thank you very much for your time, maybe if there is anything else you would like to add before we finish these interviews recommendation or anything

RT: Eeeh…other recommendation is we had a big problem with these microbiology consumables from the medical stores department, many sensitivity disks were not in the system, you can find three or four disks in the system and the rest you have to ask for a special permit to procure…., so, you see that becomes a long chain..because you have to write a letter to MSD and this causes delay in getting the consumables…and this was because these disks were not used so much and MSD wants consumables that are ordered frequently, those which are not frequently ordered are frequently not in their warehouse….

IR: So should MSD improve their ordering system for microbiology consumables?

RT: Yes, because now microbiology labs are doing a lot of work in hospitals.

IR: Okay, Thank you very much for your time and input.

RT: Okay
