# Supplementary material for: Barriers and facilitators for the implementation of Antimicrobial Stewardship Programs in Dar es Salaam Regional Referral Hospitals (RRHs)
Source: PLOS Glob Public Health. 2026 Mar 23;6(3):e0006123. doi: 10.1371/journal.pgph.0006123 (PMC13008068; doi:10.1371/journal.pgph.0006123)
Supplement: Attachment — Submitted filename: Reviewers Comments. Hope Kalasa.docx [file pgph.0006123.s004.docx]

Overall, the manuscript titled the barriers and facilitators to implementing antimicrobial stewardship programs (ASPs) in regional referral hospitals in Tanzania, addresses an important public health issue especially in settings where ASP implementation faces unique challenges. The strengths of this paper include the following:

1. The manuscript has clearly outlined the research objective and appropriate use of qualitative methodology (thematic analysis).
2. It also utilised multidisciplinary cadres (doctors, nurses, pharmacists, laboratory staff) thereby providing diverse perspectives.
3. The results are well-structured into facilitators and barriers of ASP and they are supported with illustrative participant quotes.
4. The manuscript’s discussion connects findings to existing literature at global level and sub-Saharan level which strengthens relevance.

However, it also has a few areas of improvements which if addressed can strengthen the paper. The following are some of the areas that I have identified that needs to be addressed.

1. In the abstract (Lines 15–19), the use of the word ‘efficiency’ is misleading for this paper because the study is not measuring efficiency but rather implementation experiences.
2. In the introduction, line 59-62, the paper talks about National AMR data systems that remain underdeveloped and efforts from the East Africa Public Health Laboratory Network (EAPHLN) are attempting to address these deficits. But it is not referenced for us to truly verify if these efforts are being done. A reference is needed.
3. Methods: the inclusion and exclusion criteria is not clearly defined and it is not clear if the paper only included senior staff or they also included junior staff to participate.
4. Methods: Line 128–137: the study only included 11 participants but it has not justified as to whether data saturation was reached after 11 interviews. I suggest that they clearly indicate if saturation was reached
5. Methods: Line 148 talks about a data collection tool but it not attached, I suggest that they include supplementary file for transparency
6. Results: Line 193–199 introduce themes and sub-themes with a very good structure but with only 11 participants, the generalizability of this study is very limited. I would suggest including it in the limitation section (which should be indicated) because this is a strong limitation and the findings may not be generalised beyond the study settings
7. Results: Line 204, Table summarises facilitators and barriers, but the paper could be strengthened by indicating the number of participants who supported each sub-theme (e.g., 7 out of 11 participants mentioned stockouts).
8. Discussion: The manuscript lacks a clear limitations section which can bring out critical limitation like the small sample size, potential interviewer and translation bias.
