# Supplementary material for: Barriers and facilitators for the implementation of Antimicrobial Stewardship Programs in Dar es Salaam Regional Referral Hospitals (RRHs)
Source: PLOS Glob Public Health. 2026 Mar 23;6(3):e0006123. doi: 10.1371/journal.pgph.0006123 (PMC13008068; doi:10.1371/journal.pgph.0006123)
Supplement: Attachment — Submitted filename: RESPONSE MATRIX FOR REVIEWERS.docx [file pgph.0006123.s006.docx]

| **RESPONSE MATRIX FOR REVIEWERS** |  |
| --- | --- |
| **Reviewers Comments** | **Response and action taken** |
| **Reviewer 1** |  |
| Lines 29 to 30: The sentence “Significant 30 barriers included” is a hanging statement that is not complete. | Accepted and Revised: We have removed this incomplete phrase. |
| Methods Line 126: “Strategy” should be added to the heading of “Study Population and Sampling”. | Accepted and Revised: The heading on Line 126 has been changed to “Study Population and Sampling Strategy”. |
| Line 131: The “S” in six should be a small letter. | Accepted and Revised: The capitalization of "Six" on Line 131 has been corrected to "six”. |
| Line 184: Kindly indicate the date of ethical approval was obtained. | Accepted: We have included the date of ethical approval obtained from the Muhimbili University of Health and Allied Sciences Institutional Review Board (IRB) in the revised manuscript. |
| Results Line 204: The Title of the table is too short. The authors should make the title more informative. | Accepted and Revised: The title for Table 1 ("Summary of Results") has been changed to read Summary of Themes and Sub-Themes on Facilitators and Barriers to Antimicrobial Stewardship Program Implementation in Dar es Salaam RRHs”. (Line 215-216) |
| Line 204: I also suggest the column for the theme should come first before the column for sub-themes. | Accepted and Revised: We have reformatted the table in Line 217 to place the themes before the sub theme have reformat Table 1 to place the Themes column before the Sub-themes column. |
| Line 219: The sentence “We reduced the cost of culture tests from 20,000/= to 10,000/=”. Kindly indicate the currency of the denominator. This may be converted to USD. | Accepted and Revised: We acknowledge the need for clarity. We have indicated the local currency (Tanzanian Shillings) to appropriately reflect the context. We have also added the conversion to dollars as a footnote after Line 258, ensuring the data is accessible to international readers. |
| Line 221: Be consistent; the information in bracket was italicized but in the previous line 213, it was not italicized. Same for lines 232, 237, 253. | Accepted and Revised: We have standardized the formatting throughout the results section, ensuring consistent use of italics for all participant quotes. |
| Line 241: After partners, there should be a full stop before These. | Accepted and Revised: We have corrected the punctuation. |
| Discussion Line 378: The sentence “without culture testing” should be paraphrased as “without culture and sensitivity testing”. | Accepted and Revised: We have changed the phrase to "without culture and sensitivity testing" on Line 413 to ensure precision regarding diagnostic processes essential for Antimicrobial Stewardship Programs (ASPs). |
| **Reviewer 2** | |
| Abstract (Lines 15–19): The use of the word ‘efficiency’ is misleading for this paper because the study is not measuring efficiency but rather implementation experiences | Accepted and Revised: We agree that "efficiency" is a quantitative term that misrepresents the scope of this qualitative study. We have replaced "efficiency" in the abstract and throughout the manuscript with “factors influencing the implementation of ASPs” |
| Introduction (Line 59-62): The paper talks about National AMR data systems that remain underdeveloped and efforts from the East Africa Public Health Laboratory Network (EAPHLN) are attempting to address these deficits. But it is not referenced for us to truly verify if these efforts are being done. A reference is needed. | Accepted: We have added the East Africa Public Health Laboratory Network Project (EAPHLNP) annual report that was published in 2018 to support the statement regarding efforts to combat AMR in the region. |
| Methods: The inclusion and exclusion criteria are not clearly defined, and it is not clear if the paper only included senior staff or they also included junior staff to participate. | Clarification and Revision: The study utilized a purposive sampling strategy to include healthcare professionals actively engaged in ASP-critical processes. The inclusion criteria centred on professional role and direct involvement in ASP activities, which was mentioned in Lines 138-Line141, that staff at different career stages, ranging from less than four years to more than 15 years of experience, were included. We have added more clarification on the inclusion and exclusion criteria in Lines 143-144. |
| 4. Methods (Line 128–137): The study only included 11 participants but has not justified as to whether data saturation was reached after 11 interviews. I suggest that they clearly indicate if saturation was reached. | Accepted and Revised: This is a critical point for qualitative rigor. We included a note on sample size in the limitations section (previously Line 408-Line 412). We have now explicitly added a statement to the Methods section justifying the sample size and confirming that the number of participants (N=11) was considered sufficient to achieve thematic saturation across the key stakeholder groups. |
| 5. Methods (Line 148): The data collection tool is not attached. I suggest that they include a supplementary file for transparency. | Accepted: We agree that including the interview guide enhances transparency. The semi-structured interview guide used for data collection has been included as a supplementary file labelled KII guide_English Version |
| 6. Results (Line 193–199): With only 11 participants, the generalizability of this study is very limited. I would suggest including it in the limitation section (which should be indicated) because this is a strong limitation and the findings may not be generalised beyond the study settings. | Clarification: This limitation is already acknowledged in the Study Limitations and Mitigation section (Lines 408- Line 412), noting that the sample size is small and limits generalizability beyond the regional referral hospitals in Dar es Salaam. |
| 7. Results (Line 204): The Table summarizes facilitators and barriers, but the paper could be strengthened by indicating the number of participants who supported each sub-theme (e.g., 7 out of 11 participants mentioned stockouts). | Clarification and revision: While counting frequency can indicate thematic salience, using specific counts (e.g., 7 out of 11) risks inappropriately quantifying qualitative findings derived from thematic analysis. We have revised the Results section to clearly indicate the salience and prevalence of themes using words as "consistently reported," "most participants highlighted," or "was mentioned across all three hospitals", thus strengthening the linkage without compromising the integrity of the thematic approach. |
| 8. Discussion: The manuscript lacks a clear limitations section, which can bring out critical limitation like the small sample size, potential interviewer and translation bias. | Clarification and Revision: The manuscript already includes a section titled "STUDY LIMITATIONS AND MITIGATION" starting on Line 407. We have revised and expanded this section to explicitly address potential interviewer bias, translation issues (given that interviews were conducted in Kiswahili and English), and the small sample size. |
| **Reviewer 3** | |
| General comments: There are some grammar issues especially in the use of the tenses. There are some terms that need to be explained including antimicrobial agent, antibacterial agent, antibiotic, medicines and drugs, misuse and inappropriate use, etc. | Accepted and Revised: The manuscript has undergone thorough editing to correct grammar and ensure consistent tense usage. We have clarified key terms (e.g., using Antimicrobial Stewardship Programs when referring to the program, and ensuring consistent use of antimicrobial agents or antibiotics in context) throughout the Introduction and Discussion. |
| Abstract: The authors need to align the objectives to the title (line 17). \| | Accepted and Revised**.** The objectives (Line 17) align with the title’s focus on barriers and facilitators. We have ensured that the objectives are perfectly aligned by incorporating the conceptual revision suggested by Reviewer #2 (replacing "efficiency") |
| **Abstract**: In the methods (line 20), the sentence is not clear and it needs to be paraphrased. \| | Accepted and Revised: This phrase has now been revised for improved clarity. |
| **Abstract:** In line 29-30, the sentence is incomplete. \| | Accepted and Revised: This incomplete sentence ("Significant barriers included") has been removed as noted in the response to Reviewer #1 |
| **Abstract**: In the conclusion, most of the findings would be in the results. And what were the key conclusions from the study based on the findings of the study. \| | Accepted and Revised: We recognize that the conclusion section of the abstract should synthesize the implications. We have revised the Conclusion (Lines 427-438) to focus on the overarching conclusions and recommendations derived from the findings. |
| **2. Introduction Line 56-57**: The sentence needs citation. \| | Clarification: The citation provided in Line 58 is a continuation of Line 56-57 (Now Line 55-59), which explains how these activities vary across the region and what the drivers of AMR are in the region. |
| **2. Introduction**: The authors should highlight the core concepts of ASP and the reported challenges in implementing the ASP in healthcare facilities. \| | Clarification**:** The core concepts of ASP were mentioned in Lines 86-89 (Optimizing antibiotic use, prescription audit, developing hospital formulary, standardizing prescription, and establishing therapeutic committees) |
| **2. Introduction**: In line 93-94, what is the national prevalence of AMR in the country if any? The authors should highlight to what extent, the ASP is implemented in those hospitals and nationally. \| | Accepted and Revised: We have added more references in Line 62-67 to include the burden of AMR in the country, although most data are segregated and do not provide a general rate of AMR in the country. |
| **3. Methods**: The authors should write “Methodology” as a sub-title for this section instead of “Materials and Methods”. \| | Accepted and Revised: The section heading will be changed to **“**Methodology**”** (Line 155). |
| **3. Methods**: The authors should elaborate more on the three regional referral hospitals in Dar es Salaam: Amana, Mwananyamala, and Temeke in terms of their location and capacity. \| | Accepted and Revised: The location (Dar es Salaam) and its context have been elaborated in Line 123-126. |
| 3. Methods: The authors should detail how many were males and females of these different cadres (doctors, nurses, pharmacist and laboratory staff), at the same level of training and different career stages and with work experience or have been exposed to ASP training? This may have created some bias and hence influence the outcomes. Why were administrators or managers of the hospitals not included in the study? | Accepted and Clarified: We have listed the total gender count (Six males, five females) and the cadres included (Line 143-146), noting the wide range of experience (1-15 years). Administrators were not included because the focus was on healthcare professionals actively engaging in antimicrobial prescribing, administration, and diagnostic processes, which is a core feature of ASP implementation research. Additionally, among the participants, two doctors were delegated the roles of Quality improvement officers in their hospitals, as well as the laboratory managers who take part in administrative meetings. |
| **4. Results Line 240-241**: There is a grammar issue in the sentence. \| | Accepted and Revised: We have corrected the grammatical issue identified. |
| **4. Results**: Of the selected hospital, were they experiencing the same extent of barriers and facilitators, and if not, to what extent were these in the selected study facilities? \| | Accepted and Revised: We agree that contextual variation is important. The results currently hint at differences (e.g., leadership support variation). We have revised the Results section to more systematically and explicitly discuss the variation in the extent of barriers and facilitators observed across the three specific regional referral hospitals. |
| **6. Conclusion**: The conclusions should be based on the findings of the study which is not the case for the present study. \| | Accepted and Revised. As noted previously, the Conclusion (Line 227-238) has been revised to move beyond summarizing results and focus on synthesizing the key implications and necessary actions derived directly from the study’s findings regarding facilitators and barriers. |
| **Reviewer 4** | |
| **Introduction Line 50, 54 etc**: Citation in-text should have space from text. \| | Accepted and Revised; We have adjusted the formatting to ensure proper spacing around in-text citations |
| **Introduction**: There is a minor redundancy between global AMR threat and local Tanzanian challenges. This can be linked to strengthening focus. \| | Accepted and Revised. We have streamlined the Introduction (L46-108) to ensure a smoother transition between the global AMR context, the regional context in East Africa, and the specific challenges faced in Tanzania, thereby strengthening the focus on the local implementation gap |
|  |  |
| Methods: The only limitation could be that 11 participants across 3 hospitals is small, which may lead to the risk of limited representativeness. The authors should justify why such a small study population and sampling. | Accepted and Revised: This limitation is already acknowledged in the Study Limitations and Mitigation section (Lines 408- Line 412), noting that the sample size is small and limits generalizability beyond the regional referral hospitals in Dar es Salaam. |
| **Results**: Fairly well written, however, some quotes are repetitive which I would suggest condensing. Additionally, there is no indication of variation across the three hospitals (Amana, Mwananyamala, Temeke). This would strengthen contextual understanding of the study. \| | Accepted and Revised: We have reviewed the participant quotes to condense any repetitive information. Furthermore, we have revised the Results section to explicitly highlight contextual differences and variations in barriers and facilitators observed among the three regional referral hospitals, strengthening the contextual understanding, as suggested. |
| **Discussion**: Well written. However, it may be of benefit, if it is not too descriptive (summarizing results again) rather than critically analyzing. \| | Accepted and Revised: We have edited the Discussion (L332 onwards) to reduce the descriptive summary of results and focus more on the **critical analysis** and contextualizing of our findings against international literature, highlighting similarities and differences across LMICs and HICs. |
| **Reviewer 5** |  |
| **Significance**: The study adds useful insights into ASP implementation in LMICs. However, the discussion could be strengthened by placing the findings in a broader regional/global context. Comparing these results with similar experiences in other African or Asian countries would help readers judge how generalizable the findings are. \| | Clarification and Revision. The Discussion already includes several comparisons to other LMICs and contexts, including Nepal, Kenya, Vietnam, Indonesia, Nigeria, South Africa, Ghana, and Zambia. We have revised the Discussion to make these comparisons and linkages more explicit and robust to further strengthen the regional/global context of our findings. |
| **2. Methods**: The paper would benefit from more detail about the coding process and how themes were developed. For instance, how was consistency ensured between coders, and how was saturation determined with only 11 participants? Adding a short description of the analytic steps (or even an appendix with a coding tree) would improve transparency. \| | Accepted and Clarification: The **Data Management and Analysis** section (L175) explicitly described the iterative nature of the analysis, a statement on how saturation was reached and how the small sample size was a limitation has been explained in the methodology section and in the limitation, section as requested by reviewer 2. |
| **3. Policy/Practice Relevance**: The conclusions highlight the need for resources and leadership, but they remain general. It would be stronger if the authors suggested specific actions for hospitals or policymakers (e.g., integrating ASP indicators into routine hospital reporting, improving supply chain management, or including ASP metrics in performance evaluations). \| | Accepted and Revised: We have significantly revised the Recommendation section (L439-448) to include more specific, actionable policy and practice recommendations. |
| Minor Comments: The abstract repeats some phrases between the background and objectives this could be tightened. \| | Accepted and Revised. The Abstract has been reviewed and tightened for conciseness and to eliminate unnecessary repetition of phrasing. |
| Minor Comments: The presentation of barriers and facilitators could be more structured, e.g., grouped into “system-level,” “institutional,” and “individual” factors for clarity. \| | Accepted and Clarified: The existing structure (Institutional Support, Capacity Building, Partnerships) for facilitators and (Resource/Staffing, Individual, Logistical) for barriers already aligns conceptually with System/Institutional/Individual factors. |
| Minor Comments: Some sentences in the discussion are long and could be shortened for readability. \| | Accepted and Revised: We have reviewed the discussion section for sentence length and structure to improve overall readability and flow. |
| **Reviewer 6** | |
| M1: Introduction: ASPs are defined or described multiple times (L47, L79). Keep one concise definition at the start. | Accepted and Revised: We have consolidated the definition of Antimicrobial Stewardship Programs (ASPs) to appear concisely at the beginning of the Introduction (L47-49) and ensure later mentions expand on components without redefining them. |
| M2: Introduction: Global frameworks such as **GLASS, AWaRe, and One Health** framing are not referenced when discussing surveillance gaps (L59-61). Including these will support alignment with global AMR standards. | Accepted and Revised: We have integrated key global frameworks into the discussion of AMR data systems and surveillance deficits (L421-423)) to contextualize Tanzania’s efforts within international standards. |
| M3: Introduction: The phrase in L82-83, "ASPs aim to prolong the effective life of antibiotics, improve their utilization, and **research novel antibiotics**," is speculative and misrepresents their role. Limit ASP scope to stewardship activities. | Accepted and clarified: We agree that antibiotic research is generally outside the scope of typical ASPs. We have removed the phrase **"and research novel antibiotics"** from Line 82-83 to accurately reflect the core functions of stewardship programs. |
| M4: Introduction: The transition from regional AMR context to Tanzania-specific context is abrupt (L85-86). Add a bridging sentence. | Accepted and Revised. We have added bridging sentence to smoothly narrow the focus from the regional context in Sub-Saharan Africa and East Africa (L59-67) to the specific case of Tanzania and its National Action Plan implementation. |
| Minor m3: L51-54: Avoid colloquial phrasing. Rephrase please. “make common infections untreatable” is strong and a bit conversational in tone. | Accepted and Revised. We have rephrased the statement on Line 52-53 to: **"**AMR threatens to render common infections increasingly difficult to treat". |
| Methods M1 (L130): The study includes 11 participants but provides no rationale for why this number was sufficient to achieve thematic saturation. | Accepted and Revised. We have integrated an explicit rationale in the Methods section regarding the sample size (N=11) and state that saturation was reached across key themes and stakeholder groups. |
| Methods M2 (L132): Earlier text says 'laboratory staff,' but later specifies 'three laboratory scientists, three laboratory managers,' which adds confusion. Ensure participant categories are consistent throughout. | Accepted and clarified: We will ensure consistent terminology is used for the laboratory cadres, clarifying that the participants included both scientists and laboratory managers (laboratory administrative role) |
| Methods M3 (L162): There is no description of the research team, their professional background, training, or positionality. Reflexivity is critical in qualitative research (COREQ requirement). | Clarification: Description for the research team, their academic qualifications, the role played in the research, together with their ORCID, was submitted to PLOS as part of the required information before initial submission. For reflexivity purposes, the corresponding author was in her final year of master’s degree in project management, Monitoring and Evaluation and this work was done as partial fulfillment of her degree,she was supervised by Dr. Nathanael Sirili as the main supervisor with over 50 publications in health systems in Tanzania, Mr. Luco Mwelange as co-supervisor and Ms. Margareth Mhame as the research assistant for the study. |
| Methods M4 (L181-183): Trustworthiness is mentioned generally, but key elements like intercoder reliability, audit trail, or triangulation are not detailed. | Accepted and clarified: The data management section detailed the iterative process; we have added the use of team discussions to refine interpretations (a form of intercoder review). |
| Minor m1 (L154): Provide the actual range (minimum-maximum) of interview durations instead of approximates. | Accepted and Revised. We have confirmed the actual range of interview durations and adjusted the language to reflect the true minimum and maximum range |
| Minor m2 (L148): Please inform whether the interview guide was piloted or validated before. | Accepted and Revised. We have included a statement in the Methods section clarifying how the semi-structured interview guide was piloted before formal data collection. |
| Results M1 (L224): Interpretation creeps into Results. Example: L224: “indicating the importance of supportive leadership system in implementing ASP”. Results should focus on participant data; interpretation belongs in Discussion. | Accepted and Revised. We have reviewed and omitted the phrase “indicating” in line 224. The Results section has been reviewed to ensure that interpretation is removed, and the content remains descriptive, focusing purely on participant data and thematic findings. |
| Results M3: Participant identification lacks clarity. Participant identifiers are inconsistently reported (“Lab” vs “Lab Scientist” vs. “Pharmacist”). | Accepted and Revised. We have standardized the participant identifiers used in the quotes throughout the results section. All participants from the laboratory were identified as “Lab Scientist” |
| Discussion/Conclusion M1: In the Conclusion (L393-395): The phrase: “demonstrated significant potential in reducing antimicrobial resistance and improving patient care outcomes” risks overreaching the data, as the study did not measure resistance rates or patient outcomes. | Accepted and Revised: The conclusion has been revised and rewritten to reflect the qualitative nature of our findings. |
| Minor m4: Streamline redundancy in Conclusion and Discussion. | Accepted and Revised: We have streamlined the concluding paragraphs of the Discussion and the Conclusion section to ensure the final synthesis is concise and avoids repeating content |
| Discussion/Conclusion M2 (L357): The Discussion cites CDC guidance but omits WHO GLASS, AWaRe, or One Health framing when contextualizing findings. | Accepted and Revised: We have integrated the relevant WHO global frameworks into the Discussion to contextualize the challenges and facilitators found in Dar es Salaam, especially when discussing monitoring and surveillance (L357). |
| Discussion/Conclusion M3: Study limitations are missing. | Clarification and Revision: The manuscript already includes a section titled "STUDY LIMITATIONS AND MITIGATION" starting on Line 407. We have revised and expanded this section to explicitly address potential interviewer bias, translation issues (given that interviews were conducted in Kiswahili and English), and the small sample size. |
