# Supplementary material for: Barriers and facilitators for the implementation of Antimicrobial Stewardship Programs in Dar es Salaam Regional Referral Hospitals (RRHs)
Source: PLOS Glob Public Health. 2026 Mar 23;6(3):e0006123. doi: 10.1371/journal.pgph.0006123 (PMC13008068; doi:10.1371/journal.pgph.0006123)
Supplement: Attachment — Submitted filename: MATRIX RESPONSE_2.docx [file pgph.0006123.s007.docx]

| **RESPONSE MATRIX** |  |
| --- | --- |
| **Reviewers Comments** | **Response and action taken** |
| Reviewer |  |
| Terminology consistency, the use of ASP and ASPs. Line 47 ASP was used, but line 72 ASPs was used | **Accepted and Revised**: We have reviewed the manuscript to ensure the consistent use of ASP to mean Antimicrobial Stewardship Program and ASPs where plural was used. |
| Line 136 to 141: The explanation of the study population was narrated like a result. Please paraphrase these sentences to make it sound methodological | **Accepted and Revised:** The methodology was refined and corrected as suggested by reviewer. |
| Line 187. Please insert date ethical approval; was obtained | **Clarification:** Date of ethical approval was written in line 199 under the ethical approval section. |
| Line 199, “table 1”. “T ” should be capital letter | **Accepted:** This error was corrected. |
| Line 204: The title of the table is too short. Kindly provide more descriptive narration of the table | **Clarification:** The title for Table 1 ("Summary of Results") was changed to read Summary of Themes and Sub-Themes on Facilitators and Barriers to Antimicrobial Stewardship Program Implementation in Dar es Salaam RRHs”. (Line 215-216) from the previous reviewer's comments |
| Line 241: Full stop after partners. | **Accepted and Revised:** We have added a full stop after the word "partners". |
| Line 254: full meaning of CME | **Accepted and revised** in line 270. |
| Line 269: disks, it should be discs | **Accepted and revise** in line 291. |
| Line 287: “(Participant 01, Lab)” complete the Lab ……. scientist or personnel? | **Clarification:** This was corrected from the previous review and all participants who were written as Lab...were addressed as either lab scientist or personnel. |
